# Supplementary material for: Response of Rhizosphere Bacterial Communities to Near-Natural Forest Management and Tree Species within Chinese Fir Plantations
Source: Microbiol Spectr. 2023 Jan 23;11(1):e02328-22. doi: 10.1128/spectrum.02328-22 (PMC9927156; doi:10.1128/spectrum.02328-22)
Supplement: Supplemental file 1 — Supplemental material. Download spectrum.02328-22-s0001.pdf, PDF file, 0.5 MB [file spectrum.02328-22-s0001.pdf]

**Supplementary Material**

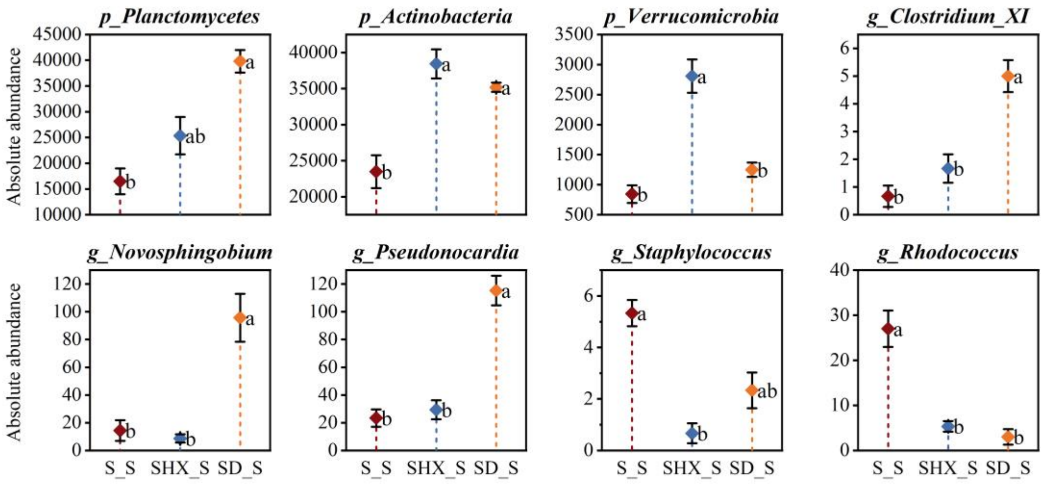


**Fig. S1** Bacterial phyla and genera with significant differences in the rhizosphere bacterial community of Chinese fir in different mixing patterns obtained by one-way ANOVA. Different letters indicate significant differences ($P < 0.05$).

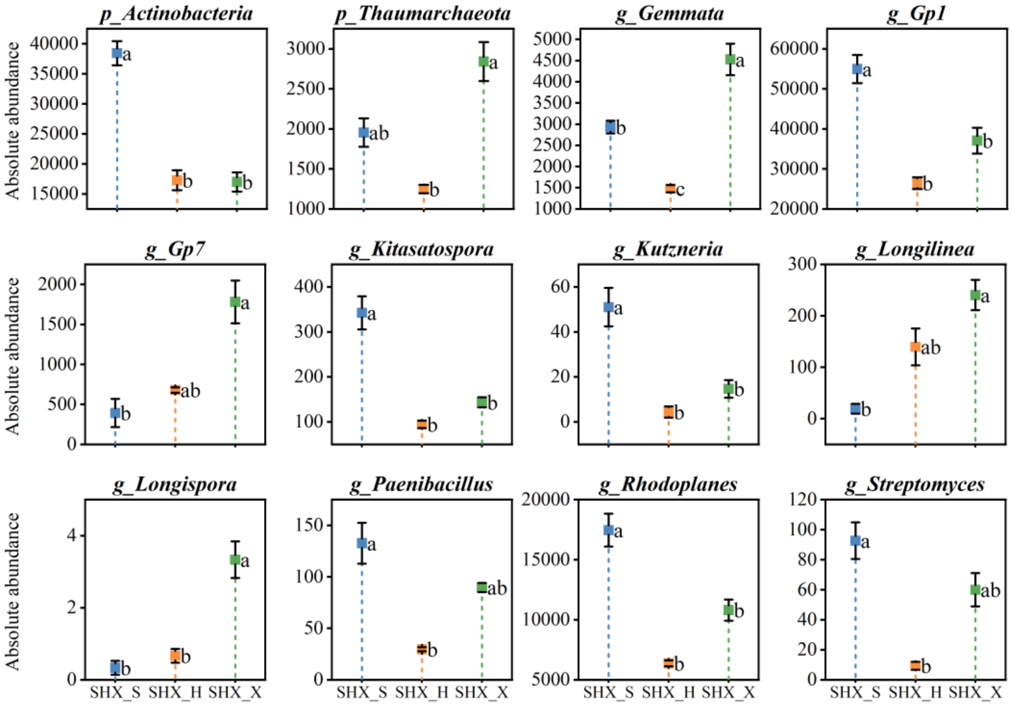


**Fig. S2** Bacterial phyla and genera with significant differences in the rhizosphere bacterial community of different tree species in SHX obtained by one-way ANOVA. Different letters indicate significant differences ($P < 0.05$).

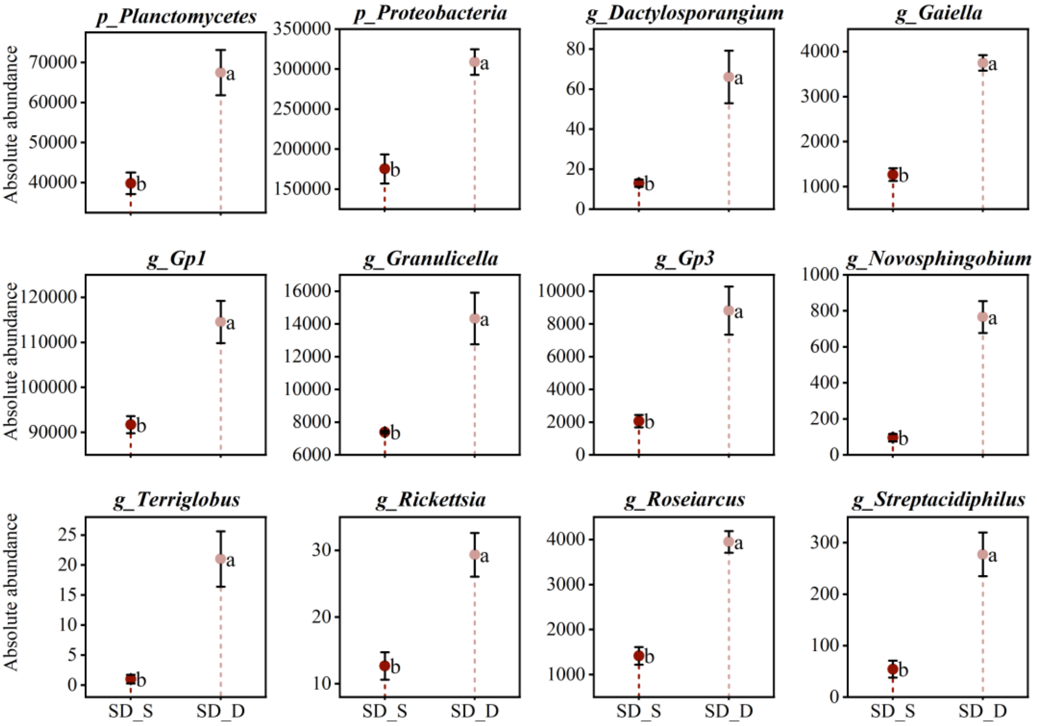


**Fig. S3** Bacterial phyla and genera with significant differences in the rhizosphere bacterial community of different tree species in SD obtained by one-way ANOVA. Different letters indicate significant differences ($P < 0.05$).

**Table S1** Bacterial network properties of rhizosphere soil in S_S, SHX_S and SD_S.

| Index | S_S | SD_S | SHX_S |
|---|---|---|---|
| Nodes | 33 | 35 | 36 |
| Edges | 59 | 43 | 47 |
| Average degree | 3.576 | 2.457 | 2.611 |
| Phylum numbers | 12 | 10 | 10 |
| Clustering coefficient | 0.822 | 0.71 | 0.866 |
| Pos/Neg edges | 58/1 | 33/10 | 38/9 |

Pos, positive; Neg, negative.

**Table S2** Bacterial network properties of rhizosphere soil in SHX_S, SHX_H and SHX_X.

| Index | SHX_S | SHX_H | SHX_X |
|---|---|---|---|
| Nodes | 36 | 33 | 32 |
| Edges | 47 | 38 | 40 |
| Average degree | 2.611 | 2.303 | 2.500 |
| Phylum numbers | 10 | 9 | 13 |
| Clustering coefficient | 0.866 | 0.858 | 0.88 |
| Pos/Neg edges | 38/9 | 29/9 | 33/7 |

Pos, positive; Neg, negative.

**Table S3** Bacterial network properties of rhizosphere soil in SD_S and SD_D**.**

| Index | SD_S | SD_D |
|---|---|---|
| Nodes | 35 | 35 |
| Edges | 43 | 55 |
| Average degree | 2.457 | 3.143 |
| Phylum numbers | 10 | 11 |
| Clustering coefficient | 0.71 | 0.732 |
| Pos/Neg edges | 33/10 | 29/36 |

Pos, positive; Neg, negative.
